# Supplementary material for: Trends of wealth-related inequality in stunting and its contributing factors among under-five children in Ethiopia: Decomposing the concentration index using Ethiopian Demographic Health Surveys 2011–2019
Source: PLoS One. 2025 Feb 12;20(2):e0314646. doi: 10.1371/journal.pone.0314646 (PMC11819561; doi:10.1371/journal.pone.0314646)
Supplement: Attachment — Submitted filename: 2. PONE-D-24-22895.docx [file pone.0314646.s001.docx]

**Review report** on paper titled “Trends of wealth-related inequality in stunting among under-five children in Ethiopia: Decomposing the concentration index using Ethiopian Demographic Health Surveys 2011-2019”

**Comments**

Title

- The authors could consider crafting a more comprehensive title that encompasses both the prevalence and explanatory variables.

**Abstract**

- In the background section, it is important for the authors to clearly establish the connection between the study's objective and the research gap, specifically addressing the need for decomposing concentration. Additionally, including confidence intervals and margins of error in the method section would be valuable.

**Introduction**

- To improve the flow of the introduction, the authors could consider starting with a discussion of the situation of stunting and then transitioning into how it can be measured and evaluated.

**Materials and methods**

- Certain explanatory variables require clarification, such as providing definitions or additional information. For example, it would be helpful to specify the meaning of "not/working" under maternal occupation and provide clear criteria for categorizing Child size at birth as large, medium, or small.
- Additionally, indicating the specific time frames for late and early initiation of breastfeeding and providing the values of the wealth index for the poorest and richest categories would enhance clarity. Furthermore, it is important to explicitly state the objectives of decomposing the relative concentration index in the methodological section.

**Report**

- Some reports looks discussion
- It is essential to exercise caution when using the wealth index and socio-demographic factors

**Discussion**

- In line 242, the conclusion is made without providing reasoning for the reduction of stunting, which may leave readers expecting further explanation.
- To facilitate a clearer understanding for drawing conclusions and making recommendations, it would be beneficial to discuss the role of each explanatory variable independently, rather than summarizing them together in line 249.

**Conclusion:**

- Given the authors' emphasis on prevalence in the report and conclusion, it would be advisable to either focus on prevalence in the title, objective, and method sections as well, or temper its significance in the results and conclusion.

**References:**

- No comments.
