# Supplementary material for: Trends of wealth-related inequality in stunting and its contributing factors among under-five children in Ethiopia: Decomposing the concentration index using Ethiopian Demographic Health Surveys 2011–2019
Source: PLoS One. 2025 Feb 12;20(2):e0314646. doi: 10.1371/journal.pone.0314646 (PMC11819561; doi:10.1371/journal.pone.0314646)
Supplement: Attachment — Submitted filename: Rebutal letter.docx [file pone.0314646.s002.docx]

Date: August 07, 2024

**To: PLOS ONE**

**Subject: Submission of Revised Manuscript**

Dear Academic Editor and Reviewers,

We are deeply appreciative of your response to our manuscript **# PONE-D-24-15084**, titled **"Trends of wealth-related inequality in stunting and its contributing factors among under-five children in Ethiopia: Decomposing the concentration index using Ethiopian Demographic Health Surveys 2011-2019**". Your valuable comments, expert suggestions, and positive evaluation have significantly enhanced the quality of our work.

In light of the constructive feedback from the reviewers, we have meticulously revised and updated the manuscript. All comments have been addressed to improve clarity and resolve the issues raised. Furthermore, an English language expert has reviewed the manuscript to correct any grammatical inaccuracies.

These revisions have greatly enriched the manuscript. We are eager to publish the manuscript in your esteemed journal to reach a relevant audience and influence policy changes aimed at eradicating stunting in Ethiopia and similar contexts.

We would like to reiterate our profound gratitude to the academic editor and reviewers for their time and constructive feedback. We have diligently addressed all the reviewer's comments, which are attached on the following page.

Best regards,

On behalf of the authors,

Corresponding author: Yawkal Tsega

Department of Health System and Management, School of Public Health,

College of Medicine and Health Sciences, Wollo University, Dessie, Ethiopia.

Email: [yawkaltsega@gmail.com](mailto:yawkaltsega@gmail.com)

Mobile: +251933559351

**Response to Reviewer 1 comments**

**Comment 1:** Data of 2016 not well discussed and information on 2019 on prevalence of stunting not consistent with 2019 EDHIS report of Ethiopia.

**Authors’ response**: Thank you for your comment and we appreciate your feedback. We made corrections regarding with discussing about EDHS 2016 data and the prevalence of stunting in 2019. We made corrections on the points raised in the revised manuscript.

**Comment 2:** In table 2 the variable region used to differentiate Agrarian, Urban and pastoralist, but the word “region” has different administrative meaning in Ethiopia. **Comment 3:** How the rural resident less than Agrarian + pastoralist?

**Authors’ response for Comment 2-3**: We appreciate your insightful comments. Indeed, you’re correct that ‘region’ refers to the administrative divisions as per the Ethiopian government. However, we employed the variable ‘administrative region’ and categorized it into Urban (comprising Addis Ababa and DireDawa city administrations), Agrarian (including Amhara, Oromia, SNNP, Tigray, and Harari regions), and Pastoralist (encompassing Benshangul Gumuz, Afar, Somali, and Gambella regions). To avoid any confusion for readers, we have operationalized these variables in the revised manuscript.

**Comment 4:** The EDHS data used in the article limited to 2011, 2016 and 2019. What was the importance of adding the other years in annexed line graph?

**Authors’ response**: Thank you for your comment and feedback. We have chosen to use the EDHS datasets from 2011, 2016, and 2019 to focus on the most recent decade. This choice allows us to analyze the most current trends in wealth-related inequality of stunting among under-five children in Ethiopia. The authors' primary analysis is focused on the recent decade, as this is the period most relevant to current policy and intervention planning.

**Comment 5:** Administrative region information constrained. While Ethiopia is the country with more administrative regions across geographic areas with varies stunting status, in EDHIS, this article provide constrained information about such variation which is not clearly discussed

**Authors’ response**: Thank you for your insightful comments. We depicted the variation in the rate of stunting among under-five children across the Ethiopian administrative regions through bar graph as presented in **Fig 1** in the revised manuscript.

**Comment 6:** Writing style, font size and type needs review for consistent presentation.

**Authors’ response**: The authors would like to express their gratitude for your expert advice. Your suggestions have been instrumental in our comprehensive revision of the manuscript's writing style, font size, and typographical errors. We have taken your recommendations into account in the revised manuscript.

**Comment 7:** There is no discussion and possible justification for increase in stunting among urban from 2016 to 2019 EDHIS

**Authors’ response**: Thank you for your comment. You’re right that discussing and justifying the increase of stunting in urban areas is crucial for health decision makers. However, the aim of our study was assessing the trends of wealth related inequality in stunting among under-five children in Ethiopia. This study didn’t assess the trends of stunting in urban area specifically.

**Comment 8:** The Example in line # 188-189 not clearly narrated

**Authors’ response**: Thank you so much for your comments and bringing this point to our attention. We addressed your suggestions and made clarity in the revised manuscript.

**Comment 9:** The conclusion not mentioned increasing trends of stunting in urban setting

**Authors’ response**: We appreciate insightful comments. The aim of this study was assessing the trends of wealth related inequality in stunting among under-five children in Ethiopia. This study didn’t assess the trends of stunting in urban area specifically.

**Response to reviewer 2 comments**.

**General comment:** Dear authors, you have researched an important topic that truly needs further exploration and intervention. Your English writing shows promise in conveying your ideas, yet could be advanced further through additional details and improved flow.

**Authors’ response**: Dear Reviewer 2, thank you for your constructive feedback and kind words about our manuscript. We appreciate your suggestion to enhance the manuscript by providing additional details and improving the flow of our writing. We certainly took these points into consideration in our revision.

**Under title**

**Comment 1:** The authors could consider crafting a more comprehensive title that encompasses both the prevalence and explanatory variables.

**Authors’ response**: We appreciate your insightful comments. We made corrections of the research topic in the revised manuscript through taking your suggestion into consideration.

**Under abstract**

**Comment 1:** In the background section, it is important for the authors to clearly establish the connection between the study's objective and the research gap, specifically addressing the need for decomposing concentration. Additionally, including confidence intervals and margins of error in the method section would be valuable.

**Authors’ response**: Thank you for your insightful comments. We agree that establishing a clear connection between the study's objective and the identified research gap is crucial. In the revised manuscript, we addressed your comments.

**Under Introduction**

**Comment 1:** To improve the flow of the introduction, the authors could consider starting with a discussion of the situation of stunting and then transitioning into how it can be measured and evaluated.

**Authors’ response**: Thank you for your comment. We revised the introduction part of the manuscript taking your comments into consideration.

**Under Materials and methods**

**Comment 1:** Certain explanatory variables require clarification, such as providing definitions or additional information. For example, it would be helpful to specify the meaning of "not/working" under maternal occupation and provide clear criteria for categorizing Child size at birth as large, medium, or small.

**Authors’ response**: Thank you for your comments and suggestions. We operationalized the some of the explanatory variables as per your suggestion.

**Comment 2:** Additionally, indicating the specific time frames for late and early initiation of breastfeeding and providing the values of the wealth index for the poorest and richest categories would enhance clarity. Furthermore, it is important to explicitly state the objectives of decomposing the relative concentration index in the methodological section.

**Authors’ response**: Thank you for your valuable suggestions. Based on your comments and suggestions, the manuscript was revised through addressing your comments and suggestions.

**Under Report/result**

**Comment 1:** Some reports looks discussion

**Authors’ response**: Thank you for your comment. We revised the overall result part of the manuscript as per your suggestions.

**Comment 2:** It is essential to exercise caution when using the wealth index and socio-demographic factors

**Authors’ response**: Dear Reviewer 2, we appreciate your suggestions. We took your suggestions into consideration in the revised manuscript.

**Under Discussion**

**Comment 1:** In line 242, the conclusion is made without providing reasoning for the reduction of stunting, which may leave readers expecting further explanation.

**Authors’ response**: Thank you so much for your comment. We revised the manuscript and addressed your comments in the revised manuscript.

**Comment 2:** To facilitate a clearer understanding for drawing conclusions and making recommendations, it would be beneficial to discuss the role of each explanatory variable independently, rather than summarizing them together in line 249.

**Authors’ response**: We sincerely value your feedback and recommendations. With your suggestions in mind, we have restructured the discussion section to include the policy implications and practical applications of our findings.

**Under Conclusion:**

**Comment 1:** Given the authors' emphasis on prevalence in the report and conclusion, it would be advisable to either focus on prevalence in the title, objective, and method sections as well, or temper its significance in the results and conclusion.

**Authors’ response**: Thank you for your comment. We revised the conclusion section of the manuscript as per your comments.

**Under References:**

**Comment 1:** No comments.

**Authors’ response**: Thank you so much for your revision on our manuscript.
