# Supplementary material for: Trends of wealth-related inequality in stunting and its contributing factors among under-five children in Ethiopia: Decomposing the concentration index using Ethiopian Demographic Health Surveys 2011–2019
Source: PLoS One. 2025 Feb 12;20(2):e0314646. doi: 10.1371/journal.pone.0314646 (PMC11819561; doi:10.1371/journal.pone.0314646)
Supplement: Attachment — Submitted filename: Rebutal letter.docx [file pone.0314646.s003.docx]

Date: November 04, 2024

**To: PLOS ONE**

**Subject: Submission of Revised Manuscript**

Dear Editor and Reviewers,

We appreciate your feedbacks to our manuscript **# PONE-D-24-15084R1**, titled **"Trends of wealth-related inequality in stunting and its contributing factors among under-five children in Ethiopia: Decomposing the concentration index using Ethiopian Demographic Health Surveys 2011-2019**". Your valuable comments, and expert suggestions have significantly enhanced the quality of our manuscript.

In light of the constructive feedbacks, we have meticulously revised and updated the manuscript for second time. Furthermore, an English language expert has reviewed the manuscript to correct any grammatical inaccuracies.

We are eager to publish the manuscript in your reputable journal, PLOS ONE, to reach a relevant audience and influence policy changes aimed at eradicating stunting in Ethiopia and other similar settings

Once again, we would like to reiterate our profound gratitude to the editor and reviewers for your time and constructive feedback. We have provided responses to all comments point by point below.

Best regards,

Yawkal Tsega

Corresponding author

On behalf of the authors

Email: [yawkaltsega@gmail.com](mailto:yawkaltsega@gmail.com)

Mobile: +251933559351

**Response to Editor’s comments**

**Comment 1:** After reviewing the revised manuscript, I strongly have some suggestion before reaching to a decision. The authors sometimes argued in the rebuttal letter that they have addressed the comment in the revised manuscript, but they didn't. My specific suggestion are given below:

**Authors’ response**: Dear Editor, thank you so much for your comments and we appreciate your feedback. We tried address all the comments in the revised Manuscript.

**Comment 2:** Comment from reviewer 1: "There is no discussion and possible justification for increase in stunting among urban from 2016 to 2019 EDHIS" - Thought this is not your primary objective, it is a significant finding. I would suggest to discuss about this change and also mention in the conclusion.

**Authors’ response**: Thank you for bringing this point to our attention. However, in this study, we did not analyse the prevalence of stunting in urban areas separately. There are no findings from this study indicating an increase in stunting in urban areas from 2016 to 2019. In fact, the prevalence of stunting among under-five children slightly decreased from 25.4% in 2016 to 22% in 2019.

**Comment 3:** Comment from Reviewer 2: "In the background section, it is important for the authors to clearly establish the connection between the study's objective and the research gap, specifically addressing the need for decomposing concentration." The author respond that they have addressed it, but I didn't notice. A clear understanding of what’s already know on this topic, what are the gaps, and how this study adds value, is very important in the background section.

**Authors’ response**: Thank you for your insightful comments. We have taken the reviewers' and your feedback into consideration and have revised the background section of the manuscript. We have improved the connection between the study's objective and the research gap, clearly establishing what is already known on this topic, identifying the gaps, and demonstrating how this study adds value. **Indicated from line number 85 to 97.**

**Comment 4:** What was your response against this comment? "Additionally, including confidence intervals and margins of error in the method section would be valuable"

**Authors’ response**: We appreciate your insightful comments. Your feedback has been instrumental in improving the quality of the manuscript. In this study, we used secondary data from the EDHS, so the margin of error is not needed. The EDHS employed a two-stage sampling method: in the first stage, enumeration areas were selected, and in the second stage, 28-30 households were chosen. Therefore, the margin of error is not required to calculate the sample size. However, we have included the 95% confidence interval in the methods statistical analysis subsection of this study.

**Comment 5:** If you have valid points, you can deny to address all the comments from reviewers. But for that you have to clearly depict your points of argument for both accepting and denying to address the comments. I strongly suggest you to read all the comment specifically and try to address them and make a rebuttal letter with your argument why you didn't address any comments.

**Authors’ response**: Authors’ response: Dear Editor, we have addressed all the comments and clarified some points. We have proofread the entire manuscript and made efforts to improve its quality.

**Response to Reviewer 2 feedback**

**Comment 1:** All comments have been addressed

**Authors’ response**: Thank you for your positive evaluation of our manuscript and we appreciate your feedback.

**Comment 2:** Dear authors, I am pleased with the quality of your work, and I believe it makes a valuable contribution to the field.

**Authors’ response**: Thank you so much for your kind words.
